# Investigation on Prevalence of Canine Trypanosomiasis in the Conservation Areas of Bwindi-Mgahinga and Queen Elizabeth in Western Uganda

**DOI:** 10.1155/2022/2606871

**Published:** 2022-09-10

**Authors:** James Robert Ochieng, Marta Planellas Bachs, Anthony Nsubuga, Innocent B. Rwego, John Joseph M. Kisakye, Laura Muro Riba, Jesus Muro Figueres

**Affiliations:** ^1^Department of Zoology, Entomology and Fisheries Sciences, College of Natural Sciences, Makerere University, Kampala, Uganda; ^2^Departament de Medicina I Cirurgia Animals. Universitat Autònoma de Barcelona, Bellaterra, Spain; ^3^Department of Plant Sciences and Biotechnology, College of Natural Sciences, Makerere University, Kampala, Uganda; ^4^Department of Biosecurity, Ecosystem and Veterinary Public Health, College of Veterinary Medicine, Animal Resources and Biosafety (COVAB), Makerere University, Kampala, Uganda; ^5^Daktari East Africa, WARM Department, Makerere University, Kampala, Uganda

## Abstract

Nowadays, despite the instauration of several control strategies, animal trypanosomiasis continues to be reported all over Uganda. Few canine African trypanosomiasis (CAT) studies have been carried out, yet dogs are known *Trypanosoma* reservoirs that share identical home ranges with livestock and serve as parasite link between livestock and humans. This study evaluates the prevalence of CAT in dogs in the Bwindi-Mgahinga and Queen Elizabeth conservation areas. This information will be useful to evaluate the possible role of dogs in the transmission cycle of *Trypanosoma* species in livestock and wild animals. Trypanosome tests using microhematocrit centrifugation/dark ground microscopy technique (MHCT) followed by conventional polymerase chain reaction (cPCR) were performed in blood samples collected from identified indigenous dogs (*n* = 124). Four (3.23%) out of 124 dogs were positive for CAT. One dog was positive with *Trypanosoma congolense* and three with *T*. *vivax*. There was no significant statistical difference in CAT prevalence rate in relation to dog's age, sex, and site (*P* > 0.05). This study reports what we believe is the first time detection of *T*. *congolense* and *T*. *vivax* in the indigenous dogs found in the Bwindi-Mgahinga and Queen Elizabeth conservation areas in western Uganda. The noticed *T*. *congolense* and *T*. *vivax* could be responsible for both canine and animal trypanosomiasis and represent a serious threat to the livestock industry. Therefore, there is a need for continuous trypanosomiasis surveillance and integrated management in contiguity to wildlife reserves.

## 1. Introduction

The African animal trypanosomiasis (AAT), commonly known as nagana, caused by protozoan parasites of the genus *Trypanosoma*, transmitted by *Glossina* spp. (tsetse fly) and mechanically by hematophagous flies in the genus *Stomoxys* (stable fly) and *Tabanus* (horse flies), is prevalent in sub-Saharan Africa [1–3]. The reservoir hosts include wild and domestic animals [1–5]. AAT caused by *Trypanosoma congolense*, *T*. *vivax*, and *T*. *simiae* is a significant constraint to livestock health and development [3, 6–9]. AAT annually affects hundreds of thousands of livestock especially cattle in sub-Saharan Africa, leading to premature abortion, infertility, and a decline in meat, milk production, and draught power [6, 7, 9–13]. The AAT dominant vectors include *Glossina morsitans*, *G*. *pallidipes*, *G*. *f*. *fuscipes*, *G. brevipalpis*, *G*. *m*. *morsitans*, and *G*. *p*. *palpalis* [1, 8, 9].

Canine trypanosomiasis has been reported in the UK [14], Brazil [15], the USA [16, 17], Senegal [18], Zambia [4], Nigeria [19], Kenya [20], and Uganda [9, 21, 22]. Studies by Abenga and Lawal [23] and Matete [20] stated that some indigenous dogs infected by trypanosomes in tsetse-infested regions of sub-Saharan Africa did not exhibit any clinical signs of the infection. Those dogs seem to be tolerant to the trypanosomes, increasing difficulties in detecting or suspecting the disease. In Uganda, numerous bovine, caprine, and porcine trypanosomiasis research have been conducted in several parts of the country [6, 7, 9, 12, 24–28]. In all these studies, reservoir hosts implicated include wildlife species such as nonhuman primates, warthogs, hippopotamus, crocodiles, and monitor lizards with little or no suspicion for dogs.

In southwestern Uganda, including Kasese, Kabale, and Kisoro districts, traditional livestock farmers keep dogs mostly for security (guarding property including livestock), herding, hunting, and scaring off vermin from the nearby protected areas. These dogs roam freely in communities, in and around Bwindi-Mgahinga and Queen Elizabeth national parks, increasing their chances of serving as a link for parasite exchange among wildlife, livestock, and humans. This is critical given the fact that Bwindi-Mgahinga and Queen Elizabeth national parks are the largest contiguous and interlinked conservation areas [29, 30] with diverse populations of protected and tourist-attractive animals including the mountain gorillas. Up-to-date, little is known about the *Trypanosoma* species parasites responsible for canine African trypanosomiasis (CAT) in indigenous dogs in Uganda. The objective of the current study was to define the profile of CAT infection burden in dogs that live in the communities contiguous to Bwindi-Mgahinga and Queen Elizabeth conservation areas. This information can increase our awareness on health and transmission risk among dogs, livestock, wild animals, farmers, and the tourists that flock the area from all over the world. On the other hand, this study will increase the knowledge of canine trypanosomiasis and the species involved in Uganda.

## 2. Materials and Methods

### 2.1. Study Sites

**Figure 1:**
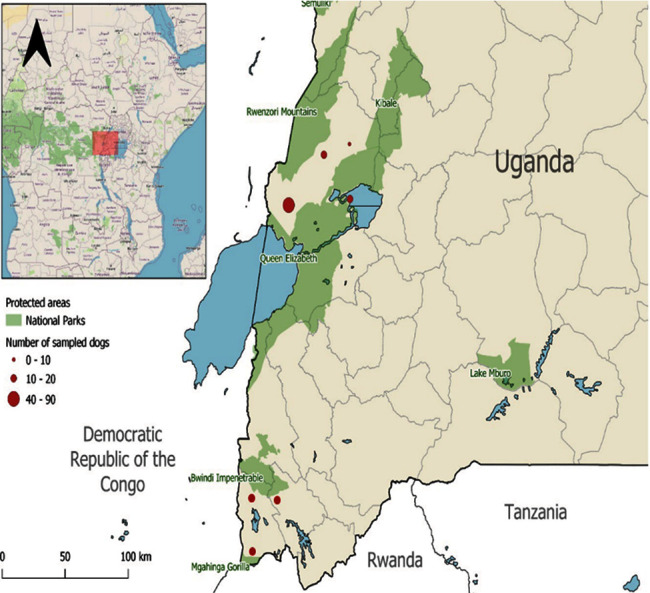
Location of western Uganda showing Queen Elizabeth, Bwindi Impenetrable and Mgahinga Gorilla National Parks, and the conservation areas (marked in round red dots) where dogs were sampled from.

Surveys were carried out in and around Bwindi-Mgahinga and Queen Elizabeth conservation area in western Uganda (Figure 1).

1. Bwindi Impenetrable National Park (331 km^2^; 0°53–1°08′N; 29°35′–29°50′E) in southwestern Uganda on the eastern edge of the Albertine Rift Valley is an afro-montane moist, evergreen rainforest with an altitude range of 1,160 to 2,607 m, characterized by steep-sided hills, peaks, narrow valleys, and average daily rainfall of 1.25 to 3.88 ml and temperature range of 19.8 to 27.7°C [31]. This “impenetrable rain forest” is one of the most diverse forest ecosystems in East Africa, with at least 223 known tree species of approximately 53% of Uganda's tree flora, and provides shelter to several birds including 23 Albertine Rift endemics, nonhuman primates, and other wild animals. Bwindi protects an estimated 400 mountain gorillas, roughly half of the world's population, including several habituated groups, which can be tracked
2. Mgahinga Gorilla National Park (MGNP), created in 1991, is small, occupying 3,390 ha (33.9 km^2^) in southwestern Uganda, bordering Rwanda to the south and the Democratic Republic of Congo (DRC) to the west. MGNP is contiguous to Virunga National Park (VNP) in the DRC and Volcanoes National Park in Rwanda. MGNP was established mainly to protect mountain gorillas (*Gorilla gorilla beringei*), vulnerable populations of indigenous plants and animals endemic to the area, and other ecological resources [32]
3. Queen Elizabeth National Park (QENP), gazetted in 1950, is located within the Albertine Rift in Western Uganda, covers a total area of 1,978 km^2^, comprised of hills, plains, forest, and swamp, and abuts the border of the Democratic Republic of Congo. QENP is the second largest National Park in Uganda, covering the districts of Kanungu, Bushenyi, Kasese, Rukungiri, Kamwenge, and Ibanda. QENP includes more than half the Uganda shoreline of the great lakes Edward and George, as well as the 20-mile-long Kazinga Channel which connects the two, and is listed as a world biosphere reserve [33, 34]

### 2.2. Sample Collection

Clearance for sampling was obtained from Uganda National Council for Science and Technology, dog owners, and district veterinary offices, in order to obtain canine blood samples during Daktari East Africa NGO activities in the conservation areas studied. Daktari East Africa (http://www.daktariandorra.org) is a veterinary NGO that, among other activities, provides rabies vaccination, deworming, treatment, and surgical sterilizations in African domestic animals including dogs. Samples were collected between the 7 and 22 July of 2018. Demographic data, signalment (species, age, sex, breed, and season), and clinical signs were recorded for each dog. According to owners' information, any dog that has never received trypanocidal treatment was included in the study. Blood samples were conveniently collected from identified indigenous dogs (*Canis familiaris*; *n* = 124), living in the conservation areas of Queen Elizabeth (13 females, 29 males), Bwindi Impenetrable (14 females, 28 males), and Mgahinga Gorilla National Park (13 females, 27 males). The dogs were aged 6 months and above and were ranked following the international veterinary age categories [35], namely, juvenile (6-12 months), young adult (aged 1 year or 12–24 months), mature adult (2-6 years), senior adults (7-11 years), and geriatric (above 11 years). Two to three milliliters of blood was obtained from the cephalic or jugular vein of each dog into a 5-ml ethylenediamine tetra acetic (EDTA) vial. Of this blood, the portion was used for the preparation of microhematocrit tubes for subsequent centrifugation and examination for trypanosomes as prescribed by [36]. The rest of the blood samples were stored in liquid nitrogen until polymerase chain reaction was performed in the laboratory.

### 2.3. Ethical Clearance

This study was approved by the Uganda National Council for Science and Technology (UNCST) on 10 June 2018. At the district level, this study was approved by the district veterinary officers (DVOs) from Kasese, Kabale, and Kisoro. Dog owners were mobilized by holding village meetings in churches and centers through the local council authorities who kept on reminding dog owners to be alert of the sampling exercises on the agreed date and time.

### 2.4. Detection of Trypanosomes with Microhematocrit Centrifugation/Dark Ground Microscopy Technique (MHCT)

The capillary tubes were centrifuged at 12,000 rpm for 5 min, and the trypanosomes present were detected by observation of parasite motion just above the buffy coat. In a sample where trypanosomes were detected, the microhematocrit tube was cut with a diamond pointed pen 1 mm below the buffy coat to include the uppermost layer of red blood cells and 3 cm above to include the plasma and then transferred to a glass slide and covered with a cover slip (22 × 22 mm). The wet smear was observed for trypanosomes under the microscope at × 10 eyepieces in combination with a × 25 objective with reduced illumination and classified into species basing on their size and movement behavior, whereby *T. vivax* is large and moves faster across the field, *T. congolense* is small in size compared to red blood cell diameter, sluggish/vibrates without progressing and its invariable attachment to red blood cells, and *T. brucei* is large and swims within a limited area [36].

### 2.5. DNA Extraction and Molecular Identification by PCR

All the collected dog samples (males =84 and females =40) were analyzed. GenElute Blood Genomic DNA kit was used to extract DNA from 100 *μ*l of each blood sample following the genomic DNA protocol (http://www.sigma-aldrich.com/genomicdna). Small aliquots of extracted DNA were stored at −20°C until required for use as template DNA for testing individual DNA extracts for infecting trypanosome species. The extracted DNA samples were subjected to a conventional polymerase chain reaction (cPCR) in 25 *μ*l reaction volumes containing 4.375 *μ*l double distilled water, 0.0625 *μ*l ITS-CF primer, 0.0625 *μ*l ITS-BR primer, 6.25 *μ*l 2xmy Taq mix enzyme, 0.25 *μ*l MgCl_2_, and 1.5 *μ*l of the template DNA. The primers (ITS-CF and ITS-BR) were designed targeting the internal transcribed spacer (ITS) region for efficient *Trypanosoma* species differentiation. The PCR cycling conditions were programmed following a protocol outlined by [37] including initial denaturation at 94°C for 5 min, followed by 35 amplification cycles each consisting of 1 min denaturation at 94°C, 1 min primer annealing at 60°C, and 1 min polymerization at 72°C, with a final extension at 72°C for 5 min. Positive controls: purified DNA of *T*. *b. brucei*, *T*. *congolense*, *T*. *vivax* and *T*. *b. rhodesiense*, and negative controls: double distilled water were included in each set of reaction. Amplified products were resolved by electrophoresis through 1.5% w/v agarose gels containing 0.5 *μ*g/ml ethidium bromide and flooded with 1× TBE buffer. Electrophoresis conditions were 55 V and 250 mA and run for 30 min. Trypanosome species determination by cPCR analysis involved comparing individual sample PCR amplicons with the corresponding band size of the positive control bands. The presence of two or more bands indicates mixed trypanosome infections, while single bands signify single/monolithic infections.

### 2.6. Statistical Analysis

Microsoft Excel spreadsheets were used for raw data entry and management. The prevalence rate was defined as the proportion of trypanosome infected dogs among all the dogs examined during the study. All analyses were carried out using IBM SPSS Statistics 23 package. Pearson Chi-square test was used to assess any statistical difference in the prevalence rate of CAT among the three conservation sites, age groups, and between the dog's sex, and *p* value of 0.05 was considered significant.

## 3. Results

The buffy coat dark ground illuminated preparations detected trypanosomes in four samples, which were also positive with the PCR. One sample had a separate band of 800 base pair (bp) for *T. congolense*, and three samples had separate bands of 250 bp for *T*. *vivax*, unlike for *T*. *brucei* of approximately 480 bp. Therefore, we conclude that four dogs out of 124 presented CAT detected by MHCT and PCR, indicating a prevalence rate of 3.23% (Table 1). The four positive dogs were three mature adult females and a mature adult male from Bwindi and Queen Elizabeth conservation areas, respectively (Table 2). They did not show evident clinical signs apart from poor body conditions and the presence of ecto-parasites mainly ticks of *Rhipichepalus* species and fleas, such as the other dogs included in the study. There was no significant statistical difference in CAT prevalence rate in relation to sex (*X*^2^ = 3.456, df = 1, *p* = 0.063), age (*X*^2^ = 8.680, df = 4, *p* = 0.07), and site (*X*^2^ = 3.494, df = 2, *p* = 0.174). There was no co-infection, and no *T. brucei* group was detected despite using ITS 1 rDNA speciation of trypanosomes targeting *T. brucei* inclusive.

## 4. Discussion

The current study reports for the first time canine African trypanosomiasis caused by *T*. *congolense* and *T*. *vivax* in free-ranging indigenous dogs that live in and around Bwindi and Queen Elizabeth conservation areas in Uganda. Animal trypanosomiasis is an important concern for livestock development allover Uganda. Trypanosomiasis is mainly reported in ruminants and pigs [9], but very little investigation has been developed in dogs [22]. The lack of knowledge about CAT in southwestern Uganda may be due to the absence of concern of a possible role of dogs in trypanosomiasis transmission, yet routine livestock treatment is carried out with different trypanocides such as diminazene aceturate (Berenil®), isometamidium chloride (Samorin®), and homidium chloride (Novidium®) in many areas [37, 38]. However, the recently recognized trypanosomes' resistance to isometamedium chloride and/or diminazene aceturate [39–41] remains a big threat to animal industry and calls for immediate satisfactory investigation. The present study offers knowledge about CAT prevalence and its possible role in transmission of trypanosomiasis among livestock, dogs, wildlife, and humans. On the other hand, the dogs studied did not manifest any clinical signs suggestive of CAT as was the same scenario in a previous related study [19]; therefore, clinical suspicion of CAT disease remains difficult. For this reason, MHCT and PCR detection can aid to define CAT incidence in an area where dogs live in close contact with livestock, wildlife, and humans. Studies in other areas evaluating canine trypanosomiasis describe clinical signs associated with the infection such as anemia, fever, body weakness, corneal opacity leading to blindness, arrhythmia, lymphadenopathy, weight loss, infertility, abortion, and death if not treated [19, 42]. One limitation of our current study was that neither thorough clinical history, nor a complete blood count and biochemistry profile of each dog was included to help define if infected dogs were clinically healthy.

All the infected dogs were mature adults which are active and may roam freely within communities and to the neighboring national parks without restriction. This could be because mature adult dogs prioritize regular exercise and/or stimulating activities including running, security, herding, and hunting, which, together with their free-roaming behaviors, exposes them to risks of diseases including CAT. However, further studies with large dog population of different age groups are still needed to define accurately the impact of age on CAT prevalence.

The 3.23% CAT prevalence rate in dogs in the current study does not vary from the 3.3% and 4.7% recorded in dogs in Buvuma Islands in Lake Victoria, Uganda [21], and Eastern Uganda [22], respectively. However, this prevalence rate is relatively low compared with the 14% and 6.42% reported in bovines in Uganda [9, 38]. This could indicate a canine resistance to infection compared to other animal species, making dogs serve as an important reservoir, providing a link for tsetse fly infection, and onward transmission to livestock and wild animals. Therefore, further studies are required in order to define a CAT prevalence and its clinical effects in all trypanosomiasis endemic and suspected areas in Uganda and Africa. The recognized *T. congolense* and *T. vivax* in the conservation areas could be due to livestock since farming is their main economic activity and the surrounding dense vegetative forests that harbor *Glossina* spp. and reservoir hosts all need to be investigated. Apparently, there are no relevant and latest entomological *Glossina* spp. data for Bwindi-Mgahinga and Queen Elizabeth conservation areas, and this should be looked at immediately. The perceived *T. vivax* may also reflect mechanical transmission by hematophagus *Tabanus* and *Stomoxys* flies which makes its control difficult even with integrated vector management as has been noticed elsewhere [3, 8, 43].

Lastly, climate change can be an important variable for CAT prevalence, but further studies are required to define its impact. The current increase in human population and changes in land use, massive penetration, and destruction of *Glossina* spp. natural conducive habitat, including wetlands, and the establishment of new contact zones might change the parasite-host interactions over time (personal observation). These factors together with global warming may trigger *Glossina* spp. and CAT mechanical transmitters to respond by survival adaptation, increasing chances of parasite-host interactions and risks of parasite spread to domestic animals.

## 5. Conclusion and Recommendation

The scarcity of CAT reports in Ugandan dogs including the studied sites can be attributed to insufficient epidemiological surveillance and trypanosomiasis control exercises on dogs unlike livestock which are routinely monitored and treated with trypanocides (personal observations). Therefore, it should be important to consider the dogs' significant role in the epidemiology of CAT. Trypanosomiasis is a big constraint in the livestock industry, and the Ugandan national policy on control of tsetse and trypanosomiasis mainly considers 5% prevalence rate of infection as the minimum for an area to be considered for appropriate intervention [39]. In the case of CAT, the recognized 3.23% prevalence rate is worrying, suggesting that careful monitoring of epidemiological data should be carried out for one to be aware of the future requirements of the interventions. The fact that even the CAT-positive dogs did not show any significant clinical signs related to trypanosomiasis infection calls for further investigation to define if there is canine resistance to this disease. There is a need for enough financial support for regular monitoring of both domestic and wild animals with molecular diagnosis and routine investigation on tsetse fly spp. and mechanical transmitters in all endemic and suspected areas in Uganda. This study increases awareness of CAT in Uganda and its relation with cattle and wild animal trypanosomiasis. It is required that further studies should screen for CAT in a larger canine Ugandan population in order to define its role and the appropriate management.

## Figures and Tables

**Table 1:** Summary showing the total number of dogs examined and their infection status in the three conservation areas.

No. of trypanosome infected dogs detected by both MHCT and PCR rDNA				
Conservation area	Sample size (n)	*T*. *vivax*	*T. congolense*	Prevalence rate (%)
Queen Elizabeth	42	0	1	2.38
Bwindi	42	3	0	7.14
Mgahinga	40	0	0	0
Total	124	3	1	3.23

**Table 2:** CAT prevalence rate (%) in relation to age in the three conservation areas.

Conservation area	Juvenile (*n*)	Young adult (*n*)	Mature adult (*n*)	Senior adult (*n*)	Geriatric (*n*)	Prevalence rate (%)
Queen Elizabeth	8	14	12	7	1	2.38
Queen Elizabeth CAT status	0	0	1 (*T. c*)	0	0	
Bwindi	10	11	11	10	0	7.14
Bwindi CAT status	0	0	3 (*T*. *v*)	0	0	
Mgahinga	13	9	7	9	2	0
Mgahinga CAT status	0	0	0	0	0	
*Total*	*31*	*34*	*30*	*26*	*3*	*3.23*

Key: *n*: number of dogs sampled; *T*.*c*: *T*. *congolense*; *T*.*v*: *T*. *vivax*.

## Data Availability

The data supporting the findings of this study are available from the corresponding author upon request.
